# Supplementary material for: Novel approach to idursulfase and laronidase desensitization in type 2 and type 1 S mucopolysaccharidosis (MPS)
Source: Orphanet J Rare Dis. 2022 Nov 3;17:402. doi: 10.1186/s13023-022-02556-7 (PMC9635105; doi:10.1186/s13023-022-02556-7)
Supplement: Supplementary file 1 — Supplementary Material 1 [file 13023_2022_2556_MOESM1_ESM.docx]

**Novel approach to idursulfase and laronidase desensitization in type 2 and type 1S mucopolysaccharidosis (MPS).**

Federico Spataro^1^, Fabio Viggiani^1^, Domenico Giorgio Macchia^2^, Valentina Rollo^1^, Albina Tummolo^3^, Patrizia Suppressa^2^, Carlo Sabba’^2^, Maria Pia Rossi^1^, Lucia Giliberti^1^, Francesco Satriano^1^, Eustachio Nettis^1^, Danilo Di Bona^1^, Maria Filomena Caiaffa^4^, Rita Fischetto^3^, Luigi Macchia^1^.

^1^Department of Emergency and Organ Transplantation, School and Chair of Allergology and Clinical Immunology, University of Bari – Aldo Moro, Bari, Italy;

^2^Department of Interdisciplinary Medicine, Clinica Medica “C. Frugoni”, University of Bari - Aldo Moro, Bari, Italy;

^3^Metabolic Diseases and Clinical Genetics Unit, Department of Pediatric Medicine, Giovanni XXIII Children's Hospital, Bari, Italy;

^4^Department of Medical and Surgical Sciences, School and Chair of Allergology and Clinical Immunology, University of Foggia, Foggia, Italy.

Corresponding Author: Federico Spataro, MD

federico.spataro@uniba.it

**ABSTRACT**

**Background.** Idursulfase and laronidase are drugs used to treat Hunter syndrome (mucopolysaccharidosis type 2) and Scheie syndrome (mucopolysaccharidosis type 1S), respectively. These are rare lysosomal storage disorders, leading to accumulation of glycosaminoglycans within lysosomes. Failure of early recognition of the disease and/or delay in starting the appropriate treatment result in severe clinical impairment and death.

For almost 20 years, enzyme replacement therapy with recombinant ~~protein~~ proteins has represented the first line therapeutic option. However, administration of idursulfase and laronidase is associated with infusion-related hypersensitivity reactions, in approx. 20% of patients.

In these patients, rapid desensitization by intravenous administration protocols has been used in order to avoid treatment discontinuation. This approach proved effective and safe. However long-term tolerance could not be achieved. Thus, we decided to combine rapid desensitization with allergen immunotherapy-like desensitization.

**Results.** Two patients with Hunter syndrome and one patient with Scheie syndrome developed severe allergy to idursulfase and laronidase, respectively, preventing them from continuing the otherwise indispensable therapy. In all three patients, the possible IgE-mediated nature of the reactions suffered was suggested by positive skin tests with the two enzymes, respectively. By devising 12-step, ~~3-dilutions~~ 3-dilution rapid desensitization protocols, we resumed the enzyme replacement therapy. However, the prolonged time required for administration (a not negligible pitfall, since therapy should be given weekly for life) and the persistent occurrence of reactions (mild but still requiring anti-allergic medication at full dosage) led us to combine rapid desensitization with a compact 11-step, 24-day allergen immunotherapy-like desensitization protocol. Thus, idursulfase and laronidase were injected subcutaneously, with a 500-fold increase from step 1 to step 11 for idursulfase and a 222-fold increase for laronidase. This strategy led to restoration of long-term tolerance, allowing weekly intravenous therapy administration under standard conditions, according to the manufacturer instructions, in the absence of side effects and with only precautionary low-dose premedication.

**Conclusion.** Rapid desensitization is a suitable and safe option in the case of idursulfase and laronidase allergy. Combination with subcutaneous allergen immunotherapy-like desensitization afforded restoration of enzyme replacement therapy given by the normal administration schedule, by inducing sustained tolerance.

**KEYWORDS**

Enzyme replacement therapy, ERT, Scheie syndrome, Hunter syndrome, drug allergy, allergen immunotherapy, AIT, rare diseases, metabolic diseases, enzyme deficiency.

**INTRODUCTION**

Mucopolysaccharidosis (MPS) type 1S (Scheie syndrome) is an autosomal recessive disease resulting from reduced function of the gene codifying for alpha-L-iduronidase. This enzyme is a lysosomal acid hydrolase, which specifically cleaves iduronic acid residues at the non-reducing terminus of long chain polysaccharides derived from dermatan sulfate and heparan sulfate proteoglycans [1]. Patients often show symptoms and clinical signs after childhood. These include wide mouth and square jaw, rhinorrhoea, sensorineural deafness, joint stiffness, mild skeletal abnormalities, carpal tunnel syndrome, mitral and aortic valve abnormalities and cervical cord compression [2,3]. Life expectancy can range from a few years, due to serious life-threatening complications leading to death in the ~~second to third~~ second-third decade, to a normal life span (albeit with significant disease morbidity) [3].

On the other hand, MPS type 2 (Hunter syndrome) is characterized by mutation of the iduronate-2-sulfatase gene (X-linked recessive transmission). In normal individuals, the iduronate-2-sulfatase enzyme catalyses the hydrolysis of C2-sulphate ester ~~bond~~ bonds of 2-O-sulfo-α-L-iduronic acid residues in dermatan sulfate and heparan sulfate [4]. Patients are generally normal at birth but develop characteristic signs and symptoms during early childhood, such as low height, abdominal hernias, ear infections, skeletal deformities, with coarse facies, a nose with a flattened bridge, an enlarged tongue. During growth, children may suffer from severe and progressive neurologic and cognitive impairment, myocardiopathy, airways obstruction. In the absence of a specific treatment, affected patients often die in the second decade of life [4,5].

Both types of MPS described here are lysosomal storage disorders that lead to accumulation of the same glycosaminoglycans, heparan sulfate and dermatan sulfate, within lysosomes. Until enzyme replacement therapy (ERT; *vide infra*) was available, there were few therapeutic options that could have a significative impact on the natural course of both diseases. ERT consists of slow intravenous administration of laronidase, in MPS type 1S, and idursulfase, in MPS type 2 [6]. Both enzymes are obtained by recombinant DNA technology. Thus, laronidase ~~compensate~~ compensates for the reduced enzyme function in MPS type 1S [7], whereas idursulfase compensates for the more severe deficiency found in MPS type 2 [6]. However, administration of laronidase and idursulfase is frequently associated with infusion-related hypersensivity ~~reaction~~ reactions (HSR), in most cases represented by flushing, erythema, skin rash, urticaria, pruritus [7,8]. In rare cases, severe anaphylaxis may occur, possibly leading to treatment discontinuation.

In patients with a history suggestive of severe anaphylaxis to laronidase and idursulfase, respectively, ~~a~~ rapid desensitization ~~approach has~~ and similar approaches have been attempted and ~~has~~ have proved effective in maintaining ERT, avoiding the discontinuation of the treatment [9,10,11]. Rapid desensitization protocols consist in the induction of a temporary state of tolerance to a specific drug responsible for a given hypersensitivity reaction. This is achieved by administering increasing doses of the offending medication in a non-linear manner, over a period of time longer than the standard one, until the total cumulative therapeutic dose is given and tolerated. It is a delicate and moderately risky procedure, used only in patients in whom therapeutic alternatives are not available or significantly less effective. Desensitization is mainly performed in IgE-mediated reactions, but also in reactions ~~where~~ in which drug-specific IgE have not been demonstrated. Despite its clinical success, little is known about the ~~mechanisms~~ mechanism and molecular targets of drug desensitization. Mast cells and basophils seem to be targets in the process, since mediators from these cells are released during hypersensitivity reactions to drugs, as well as during desensitization procedures [12]. Generally, rapid desensitization protocols consist of 12 consecutive steps (usually, using 3 solutions with increasing drug concentrations). At each step, the rate of drug administration is increased by 2-fold to 2.5-fold, with a 15’ duration for each step, apart for Step 12, which lasts about 3 ~~hours~~ h [13].

Notably, the rapid desensitization protocols developed so far for MPS require up to 6 ~~hours~~ h, for ERT administration, and should be performed every week, for life. Moreover, in spite of careful management and appropriate premedication, sometimes, mild, amenable HSR still occur [12].

On the other hand, IgE-mediated allergic diseases have been treated for more than 110 years by allergen immunotherapy (AIT) [14], given subcutaneously (sublingual AIT was also developed in the 90’). Subcutaneous AIT is regarded as effective and safe in the desensitization of patients with pollen-related or house-dust mite-related respiratory allergy [15,16] and, particularly, in Hymenoptera venom allergy [17,18,19]. Usually, subcutaneous AIT comprises an induction phase, in which increasing doses of the culprit allergen are administrated at short intervals of time (e.g, weekly), and a maintenance phase, in which a fixed dose of allergen is administrated at relatively larger intervals of time (e.g, monthly) [20,21]. Usually, the maintenance dose is approx. thousand-fold higher than the first induction-phase dose [22].

Here, we report on three MPS patients (one case of MPS type 1S and two cases of MPS type 2) who developed severe HSR and discontinued ERT. All three patients were desensitized by a protocol designed according to Castells [13]. However, having resumed ERT, we ~~proceed~~ proceeded further with a novel AIT-like (allergen immunotherapy-like) ~~11-steps~~ 11-step desensitization approach, requiring three weeks, which ensured long-lasting tolerance, allowing safe continuation of ERT by a simplified administration schedule. To our knowledge this is the first report of AIT-like desensitization for ~~MPS ERT~~ allergy to ERT in MPS.

**METHODS**

**Patients.**

We report, in chronological order of observation, on three male patients with MPS, who developed severe HSR to ERT. They were seen and desensitized between 2019 and 2022, at the Allergology Unit of University Hospital of Bari (Italy), in cooperation with the Unit of Paediatric Metabolic Disease and Medical Genetics and the Rare Disease Unit. The three patients were aged 38 (~~Patient~~ patient 1; MPS type 2), 35 (~~Patient~~ patient 2; MPS type 1S) and 9 (~~Patient~~ patient 3; MPS type 2), respectively.

**ERT medication.**

Idursulfase (Elaprase®), manufactured by Shire Pharmaceutical Ireland Ltd, Dublin, Ireland, was obtained from Shire Human Genetics Therapies AB, Stockholm, Sweden, as a clear solution (2 mg/ml), and was kept at ~~4°C~~ 4 °C until used [23].

Laronidase (Aldurazyme®), produced by BioMarin Pharmaceutical Inc., Novato, USA, was provided by Genzyme Ireland Ltd., Waterford, Ireland, also as a clear solution (100 U/ml – 0.58 mg/ml), kept at ~~4°C~~ 4 °C [24].

**Skin tests.**

The three patients were subjected to skin prick test (SPT) and intradermal test (IDT), with ~~idursufase~~ idursulfase (patients with MPS type 2) and with laronidase (patient with MPS type 1S), respectively. Idursulfase was ~~used as undiluted drug~~ used undiluted (2 mg/ml) for SPT, ~~while it was diluted 1000-fold (0.002 mg/ml), 100-fold (0.02mg/ml) and 10-fold (0.2 mg/ml) with saline solution~~ while for IDT it was diluted 1,000-fold (0.002 mg/ml), 100-fold (0.02mg/ml) and 10-fold (0.2 mg/ml), respectively, with saline. Laronidase was tested undiluted (100 U/ml or 0.58 mg/ml) for SPT, while it was diluted 100-fold (1 U/ml or 0.0058 mg/ml) and 10-fold (10 U/ml or 0.058 mg/ml) with saline for IDT. The leaflets of the two medications can be downloaded at the sites below: <https://www.ema.europa.eu/en/documents/product-information/elaprase-epar-product-information_en.pdf> (Elaprase, idursulfase); <https://www.ema.europa.eu/en/documents/product-information/aldurazyme-epar-product-information_en.pdf> (Aldurazyme, laronidase). Skin tests were performed in a quantitative way [25]. Histamine (10 mg/ml for SPT and 0.002 mg/ml for IDT) and saline were used as the positive control and the negative control, respectively.

**Lymphocyte Proliferation Test (LPT).**

Two venous blood samples (15 ml unclotted with EDTA 2 µM and 15 ml clotted) were obtained from two of three patients (patient 2, with MPS type 1S, and patient 3, with MPS type 2), in order to perform LPT [26]. Briefly, upon plasma removal and suspension of the blood cellular moiety in Dulbecco’s phosphate buffered saline (PBS), peripheral blood mononuclear cells were isolated by gradient centrifugation (800 x g, 45’) on Lympholyte® (Cedarlane, EuroClone, Milan, Italy). Mononuclear cells were maintained in Dulbecco’s modified Eagle’s medium (form Sigma, Milan, Italy), with 10% autologous serum (v/v; ~~form~~ from the homologous clotted sample), with streptomycin (100 μg/ml), at 37°C, in a 5% CO_2_, vapour-saturated atmosphere, in 64 cm^2^ glass Petri dishes, for 4 days, in order to allow clearance of the monocyte-macrophage component. At day 5, micro-cultures were generated with the resulting purified lymphocytes (6 × 10^4^ cells in 200 μl), in ~~the same~~ fresh buffer as above.

The micro-cultures of purified lymphocyte were incubated in triplicate with the two drugs of interest (idursulfase and laronidase), respectively, at three different ~~ten-fold~~ 10-fold concentrations: the therapeutic concentration (TC; calculated on the basis of the drug distribution volume); TC/10 and TCx10 (defect concentration and excess concentration, respectively). Triplicate micro-cultures incubated with phytohemagglutinin-M (from *Phaseolus vulgaris*; 2.25 μg/ml; also form Sigma) and the medium alone served as the positive and the negative control, respectively. The distribution volume and the TC ~~were 198 ml/kg and 24 mg, respectively, for idursulfase~~ assumed for idursulfase were 198 ml/kg and 2.9 ml/l [27]. The distribution volume and the TC ~~were 0.44 l/kg and 6,800 U (39.44 mg), respectively, for laronidase~~ assumed for laronidase were 0.60 l/kg and 162 U/l (0.940 mg/l) [24].

Following ~~four days~~ a 4-day incubation with the drug, lymphocyte proliferation was assessed upon inclusion of the non-radioactive thymidine analogue 5-bromo-2’ deoxyuridine (BrdU; 100 μM), in the micro-cultures, for 2 h. Then, we assessed the incorporation of the nucleotide in proliferating cells by an anti-BrdU monoclonal antibody (7.5 U/ ml; from Roche Diagnostics GmbH, Mannheim, Germany) [28]. LPT was deemed positive, when the proliferation rate of any of the three concentrations tested compared to the negative control (stimulation index) equaled or exceeded 2 [29,30].

**Rapid Desensitization.**

For both drugs (idursulfase and laronidase), rapid desensitization was performed preparing three different solutions for intravenous administration: for idursulfase, a mother solution (0.048 mg/ml), a 1/10 dilution (0.0048 mg/ml) and a 1/100 dilution (0.00048 mg/ml); for laronidase, a mother solution (0.079 mg/ml), a 1/10 dilution (0.0079 mg/ml) and a 1/100 dilution (0.00079 mg/ml). The desensitization protocol consisted of 12 consecutive steps with increasing speeds and doses, at each dilution/bag. Each step lasted 15 minutes, apart for step 12 which lasted approx. 180 minutes. Altogether, the drug infusion lasted almost 6 ~~hours~~ h. In Table 1 and Table 2, the rapid desensitization protocols for idursulfase and laronidase, respectively, are shown.

**AIT-like ~~desensitizazion~~ desensitization.**

AIT-like desensitization was performed through subcutaneous injections of idursulfase, for the two patients affected by MPS type 2 (patient 1 and patient 3), and laronidase, for the patient affected by MPS type 1S (patient 2). The subcutaneous AIT-like desensitization protocol consisted of 11 steps, with a total 3-week duration. Each step was carried out every other day, consisting in 3 or 4 subcutaneous injections of the offending drug, spaced 20 minutes apart, at each session. For idursulfase, step 7 of the protocol was repeated ~~four~~ 4 additional times (up to step 11). For laronidase, step 8 was repeated three additional times (up to step 11). In Table 3 and Table 4, the AIT-like desensitization protocols for idursulfase and laronidase, respectively, are shown.

AIT-like desensitization for idursulfase was carried out by using the follow concentrations: idursulfase diluted 100-fold (0.02 mg/ml); idursulfase diluted 10-fold (0.2 mg/ml); undiluted idursulfase (2 mg/ml). Between step 1 and steps 8-11, there was 500-fold increase in the dosage administered.

As for the less concentrated laronidase, AIT-like desensitization was carried out by using the ~~follow~~ following concentrations: laronidase diluted ~~100 folds~~ 100-fold (1 U/ml); laronidase diluted 10-fold (10 U/ml); laronidase undiluted (100 U/ml); and finally, laronidase concentrated ~~5 folds~~ 5-fold (500 U/ml). The difference between step 1 and steps 7-11 was 222-fold.

Laronidase was ~~concentred~~ concentrated 5-fold by ultrafiltration. Ultrafiltration devices (Centricon, Amicon; 10,000 Da MW ~~cut off~~ cutoff) were from Millipore, Bedford, MA, U.S.A. The devices of choice allowed separation of molecules above 10 kDa, found in the retentate, from molecules below 10 kDa, collected in the eluate. Thus, two ml of laronidase were loaded into the ultrafiltration device and subjected to centrifugation, at 3,000 x g, for 20 minutes at 4 °C. At the end of the centrifugation, about 500 µl of concentrated laronidase were collected in the upper chamber of the device. The concentrated drug (retentate) was then sterilized by filtration (0.22 μ syringe driven filters, Millex, Millipore), under ~~aseptical~~ aseptic conditions.

**RESULTS**

**Case presentation.**

**Clinical features****.** Three MPS cases were studied. Here, they are presented in chronological order of observation. A 38-year-old patient (patient 1) was diagnosed with Hunter syndrome, at age 6. Only at age 30, the patient could be treated with ERT (idursulfase), 24 mg, given weekly intravenously in 1-3 h. This therapeutic approach was initially well tolerated. However, after one year of treatment, he developed urticaria localized particularly at the upper limbs, approx. 90’ after beginning ERT infusion ~~therapy~~. The treatment was then discontinued, until June 2019, when the patient, then 38, restarted the weekly infusions of idursulfase (24 mg), at the Rare Disease Unit of Bari University Hospital. The first 6 administrations were tolerated without side effects. However, during the seventh infusion, he developed again generalized urticaria. The infusion was halted and hydrocortisone (500 mg) and chlorpheniramine (10 mg) were administered, with resolution of symptoms. No epinephrine was required. The patient was then referred the Allergic Disease Unit at the same Hospital.

In consideration of the anamnesis (repeated urticarial events, immediate nature of the reactions, ~~promp~~ prompt response to anti-allergic treatment), a case of immediate allergy to idursulfase was suspected and a thorough ~~allergological~~ allergy workup was performed, with the purpose of desensitizing the patient, in order to avoid further discontinuation of ERT. Thus, we performed SPT and IDT. SPT, were performed by full-strength idursulfase solution (2 mg/ml) and proved negative. For IDT, we used increasing idursulfase concentrations: 0.002 mg/ml, 0.02 mg/ml, 0.2 mg/ml, respectively. The tests proved positive. While 0.002 mg/ml concentration produced no reaction, the 0.02 mg/ml idursulfase concentration yielded a wheal of 10 mm (average diameter), deemed as positive. According to the protocol adopted [25], IDT with 0.2 mg/ml concentration was not performed (due to the 10 mm diameter wheal obtained with the 0.02 mg/ml concentration).

LPT could not be performed, in this case. Since the skin test results were suggestive of immediate-type hypersensitivity, considering the absolute necessity of maintaining ERT, we devised and implemented a 3-bag, 12-step rapid desensitization protocol, according to Castells [13]. Thus, we used 3 idursulfase dilutions at increasing concentrations: 0.00048 mg/ml, 0.0048 mg/ml, 0.048 mg/ml. The weekly target dose was 24 mg, given intravenously (calculated on the patient body weight). The rapid desensitization protocol is reported in Table 1. The procedure was implemented for 8 consecutive times, with no adverse events, with the exception of the first infusion, when at step 12~~, infusion rate 150 ml/h,~~ (infusion rate 150 ml/h) mild itchy urticarial lesions were observed at the level of the axillary spaces, the chest and abdomen, treated with chlorphenamine 10 mg and hydrocortisone 500 mg, intravenously. The infusion rate was reduced to 40 ml/h for approx. 60 minutes and then gradually brought back at 150 ml/h. The procedure was finalized, without further adverse reactions.

Having resumed ERT, we sought to restore immune tolerance by an 11-step AIT-like desensitization protocol. While continuing the weekly intravenous administrations of idursulfase, according to the rapid desensitization protocol described, idursulfase was also administered subcutaneously every second day, with increasing concentrations of the offending drug, for 11 consecutive times, starting from 0.008 mg at step 1- day 1 up to 4 mg at step 7- day 14, with a 500-fold increase (Table 3). Notably, 4 mg represented 17% of the whole weekly dose and was readily tolerated. Step 7 was then repeated 4 more times, up to step 11- day 26. Dosages at each step of the AIT-like protocol were given fractionated by three subcutaneous injections, from step 1 to step 4, and by 4 injections, from step 5 to step 11. Single ~~njections~~ injections were given 20’ apart from each other and were performed at the level of the external side of the arm, like in the classic subcutaneous AIT. Sessions lasted from 90’ to 120’, including 30’ of final observation for possible side effects. At steps 1 and 2, idursulfase was diluted 1:100 (0.02 mg/ml). At steps 3 and 4, the drug was diluted 1:10 (0.2 mg/ml). From step 5 to 11, the drug was used undiluted (Table 3). The total dose administered at the end of the procedure (day-26) was 23.58 mg (almost equivalent to the weekly intravenous target dose of 24 mg). Overall, the desensitization procedure was well tolerated. No side effects, either immediate or delayed, local or general, were recorded. No premedication was required, throughout the protocol. Upon completion of the AIT-like desensitization procedure, idursulfase was delivered on a weekly basis for 22 months, according to a simplified schedule: *i.e*., full-strength concentration (0.048 mg/ml; 24 mg in 500 ml saline), at a rate of 150 ml/h for approx. 3 h. After completion of the AIT-like course, anti-allergic premedication was ~~deemed not further necessary, apart for the first two infusions, when fexofenadine 180 mg was given orally, 1 h prior of the ERT~~ reduced to fexofenadine 180 mg given orally the night before ERT. For the first two infusions, fexofenadine 180 mg was given orally also 1 h prior of ERT. No adverse events were recorded, with this simplified schedule. In this case, due to circumstantial reasons, no AIT-like maintenance injections were given (see below).

Patient 2. A 35-year-old farmer was diagnosed with Scheie syndrome at age 13. The patient was treated with weekly administrations of laronidase since diagnosis, at the Metabolic Diseases Unit of Giovanni XXIII Bari University Hospital. Thus, 39.44 mg (6,800 U) of laronidase, diluted in 250 ml saline, were given by slow intravenous infusion in about 6 h (the infusion rate was increased from 5 ml/h up to 50 ml/h every 15’ and then kept at 50 ml/h till the end). This therapeutic approach was well tolerated until July 2021, when the patient developed diffuse urticaria and ~~lips~~ lip angioedema, 10’ after starting the infusion. The treatment was suspended and corticosteroids and antihistamines were administered, with resolution of symptoms. The patient was then referred to the Allergy Unit of the same Hospital. A thorough ~~allergologic~~ allergy workup was performed with the purpose of desensitizing the patient, in consideration of the need for avoiding discontinuation of laronidase therapy. Thus, we performed SPT and IDT with laronidase. For the SPT, we used full-strength solution of laronidase (100 U/ml; 0.58 mg/ml). For the IDT, we used increasing concentrations of laronidase: 0.0058 mg/ml (dilution 1:100) and 0.058 mg/ml (dilution 1:10). While SPT were negative, IDT were positive at both 1:100 dilution and 1:10 dilution, with average wheal diameter of 7 mm and 8.5 mm, respectively. Furthermore, LPT with BrdU assay was performed, with a negative result, adding to the possible immediate-type nature of the allergic reactions suffered. Given the need of continuing the ERT, in consideration of the clinical history and the results of the skin tests (suggestive of immediate hypersensitivity) we ~~deviced~~ devised and implemented a 3-bag, 12-step protocol of rapid desensitization. The target dose was 39.44 mg – 6,800 U, intravenously (calculated on patient body weight). Thus, we used 3 laronidase dilutions at increasing concentrations: 0.00079 mg/ml – 0.136 U/ml (in 100 ml saline), 0.0079 mg/ml – 1.36 U/ml (in 100 ml saline), 0.079 mg/ml – 13.6 U/ml (in 500 ml saline). The procedure lasted 5 h and 47’and was performed 4 consecutive times. The rapid desensitization protocol is reported in Table 2.

At step 11 of the first infusion, the patient developed severe generalized urticaria and angioedema of the lips. We administered hydrocortisone (100 mg) and chlorpheniramine (10 mg) intravenously and, in the absence of a prompt resolution, adrenaline, ~~0,5~~ 0.5 mg intramuscularly, leading to immediate remission. The infusion was stopped. However, the desensitization procedure was repeated the next day. This time, without any side effects, upon premedication with betamethasone 2 mg and fexofenadine 180 mg, per os, the night before, at 08:00 p.m., and chlorphenamine 10 mg and dexamethasone 4 mg, intravenously, just before starting the procedure.

During the following three desensitization procedures, ensuring the weekly ERT administration, no adverse effects occurred. Premedication with dexamethasone and chlorphenamine, at the same dosage as above, was given at the beginning of the rapid desensitization procedure.

Moreover, in order to induce long-lasting immune tolerance, also in consideration of the fact that the weekly ERT had to be given for life, an 11-step AIT-like desensitization protocol was devised and implemented (Table 4), rather similar to that previously adopted for idursulfase ~~(Table 4)~~. The protocol foresaw 4 daily subcutaneous injections, given 20’ apart from each other, at any of the 11 steps, every second day. Increasing concentrations of laronidase were used: 0.058 mg/ml (10 U/ml) at steps 1 and 2; 0.58 mg/ml (100 U/ml) at steps 3 to 6; 2.9 mg/ml (500 U/ml) at steps 6 to 11 (Table 4). Thus, at step 1- day 1, 9 U of laronidase were injected. While 2,000 U were given at step 8 - day 16 (222-fold increase). Notably, 2,000 U corresponded to 34% of the weekly dose. The 2,000 U dose was then repeated three more times up to step 11- day 23. Altogether, 10,400 U were administered at the end of the procedure. No adverse reactions occurred. No premedication was required. ~~The AIT-like desensitization protocol is reported in Table 4~~**.** After completion of the AIT-like desensitization procedure (Table 4), we resumed giving laronidase by intravenous administration, on a weekly basis, reducing both infusion time and premedication. Thus, the patient tolerated the weekly laronidase ERT according to the standard protocol adopted before the emergence of the first HSR: full-strength concentration (0.157 mg/ml - 27.2 U/ml; 39.44 mg - 6,800 U, in 250 ml saline), with 5 steps at increasing rate of administration (10 ml/h, 20 ml/h, 40 ml/h, 80 ml/h, 100 ml/h, respectively). The first 4 steps lasted 15’ each, while the fifth one lasted 127’. The procedure was completed in 3 h and 7’. Premedication consisted ~~in~~ of antihistamines only, particularly oxatomide 30 mg per os, the night before and 30’ before the infusion, and chlorphenamine 10 mg intravenously between step 4 and step 5 of the infusion. Moreover, in order to maintain the immuno-tolerance state, we administered 2,000 U of 5-fold concentered laronidase subcutaneously (4 subcutaneous injections of 1 ml), once-a-moth, as for the classic AIT.

Under these conditions, when this report was written, the standard weekly infusion protocol had been performed at home for 8 months, without any adverse events.

Patient 3. A 9-year-old boy was diagnosed with Hunter syndrome in 2017 at the Metabolic Disease Unit of Giovanni XXIII Pediatric Hospital of Bari. Thus, he started the weekly ERT with idursulfase. Twenty-four mg of idursulfase, in 250 ml saline, were given at home by slow intravenous infusion in about 4 h (the infusion rate was approx. 60 ml/h). ERT was well tolerated till July 2021, when the boy developed diffuse urticaria during the last minutes of infusion (no premedication had been administered). The same reaction occurred at the successive infusion. On both occasions antiallergic therapy was given with resolution of symptoms. However, home ERT was discontinued and the subsequent administrations of idursulfase were performed at the Metabolic Disease Unit. During the first two ERT infusions the patient developed urticaria and dysphonia. Corticosteroids and antihistamines were administrated with symptom resolution. But the full dosage ERT administration was interrupted. The idursulfase dosage was then lowered to only 4 mg, tolerated for 5 moths, up to December 2021, when, due to deterioration of the clinical conditions, the boy was referred to our Allergy Clinic. An ~~allergological~~ allergy workup was carried out with the purpose of restoring the weekly full 24 mg idursulfase dosage by implementing a rapid desensitization protocol.

A blood sample of the patient was sent to Geneva-Meyrin, Switzerland, for anti-idursulfase IgE assessment. The assay proved negative.

Thus, we performed SPT and IDT with idursulfase. While SPT yielded a negative result, IDT were positive at both 1:100 and 1:10 dilution, with an average wheal diameter of 7 mm and 9 mm, respectively. LPT with BrdU assay was also performed, with a negative result.

In consideration of the clinical history and the results of the skin tests, suggestive of immediate hypersensitivity, and the need of continuing the weekly ERT, we implemented a 3-bag, 12-step protocol of rapid desensitization, according to Castells. The target dose was also in this case 24 mg, intravenously (calculated on patient body weight). Thus, we adopted the same rapid desensitization protocol as for patient 1 (Table 1).

At the first infusion, premedication given to the patient was: cetirizine 7.5 mg, orally, the night before the infusion, and chlorphenamine 5 mg intravenously, 30’ before the infusion. Moreover, 5 mg of chlorphenamine were given between step 8 and step 9. However, during step 12, the little patient developed severe ~~general~~ generalized urticaria. The infusion rate was lowered and hydrocortisone, 200 mg intravenously, and cetirizine, 5 mg orally, were given with resolution of symptoms. The 150 ml/h infusion rate was then resumed ~~to~~ and the ERT was completed.

Because of this reaction, a week later, the second rapid desensitization procedure was carried out according to a modified 4-bag, 20-step protocol and, on the same day, AIT-like desensitization was started (day1- step1), performed according to the protocol implemented for patient 1 (Table 3). The whole AIT-like desensitization cycle was completed about one month later, (step 11; Table 3). Meanwhile, the first 20-step rapid desensitization procedure again was accompanied by generalized urticaria, requiring chlorphenamine and flow rate reduction (in spite of ~~this~~ that, the procedure was anyway completed). However, the successive two intravenous infusions (modified 20-step procedure), overlapping the subcutaneous AIT-like injections of idursulfase, were successfully completed without HSR and ~~requiring~~ required neither supplementary medication nor flow rate lowering. Thus, with the next 5 weekly intravenous infusions, we rapidly decreased the number of bags and step, and consequently the time required for administering ERT, down to only one solution strength (24 mg/250 ml saline; 0.096 mg/ml), given in 6 steps, in approx. 2 h (10, 20, 40, 80, 150, 180 ml/h, with increase every 15’, apart from the last step which lasted approx. 1 h).

At the time when this manuscript was written, the weekly simplified ERT protocol had been administered 12 times without any HSR and with only a light premedication (cetirizine 7.5 mg, orally, the night before and 30’ before the infusion). The AIT-like desensitization was maintained with a monthly 4 mg dose of idursulfase, given subcutaneously (to date, for 5 months).

**DISCUSSION**

MPS are various and rare metabolic disorders, which can lead to progressive clinical impairment and death, mostly if diagnosis is not done during childhood and/or ERT, the first line therapy for these patients, is not promptly started. Life expectancy can range from a few years to a normal life span, depending on type, severity of the MPS and therapy. ERT has significantly changed the natural history of these diseases and patients’ life expectancy. However, its real-world impact is limited for at least three negative reasons: a) costs are exceedingly high and, therefore, difficult to afford in low-income countries; b) ERT should be given on a weekly basis for life, by usually complex intravenous administration schedules; c) allergic reactions are frequent (up to 20% of cases) ~~[30,31]~~ [31,32], given the non-self nature of these protein enzymes, obtained by recombinant DNA technology.

Here, we report on three male patients with MPS type 2 (patient 1 and patient 3) and MPS type 1S (patient 2), who were subjected to ERT with idursulfase and laronidase, respectively, and developed immediate severe HSR during the administration of these drugs, leading to ERT discontinuation.

In fact, HSR during idursulfase and laronidase infusions occur frequently [31,32]. In these three patients, in consideration of the importance of maintaining ERT and the severity of HSR suffered, which precluded continuation of the therapy by simply slowing the flow rate or potentiating the antiallergic premedication, rapid desensitization protocols, according to Castells, were devised and implemented [13].

Rapid desensitization procedures by intravenous infusion have proved effective and safe and have been employed in the management of allergy to a variety of drugs, such as chemotherapeutic drugs, antibiotics, monoclonal antibodies, iron therapy, etc [33-45].

Rapid desensitization has been successfully performed mainly for chemotherapeutic drugs, such as platins, taxanes, doxorubicin, and in 94% of cases only mild or no reactions occurred [13].

Different antibiotics were administered successfully by rapid desensitization schedules, such as penicillin G, in patients with syphilis [33,34], and ceftazidime/avibactam in klebsiella pneumoniae severe respiratory infection [35]. Moreover, the literature abounds of case reports, such as the successful 4-bag/16-step rapid desensitization for ceftriaxone performed in 17-year-old patients, also affected by ~~mastcell~~ mast cell activation syndrome [36].

HSR are also frequent during monoclonal antibody therapy and rapid desensitization procedures proved to be effective and safe for rituximab [13,37], tocilizumab [38], infliximab [39], trastuzumab [40]. As for, iron therapy, rapid desensitization schedules were published by our group and others [41,42]. Moreover, rapid desensitization has been used also in the case of allergy to recombinant enzymes in rare metabolic diseases, including allergy to ~~Pegvaliase~~ pegvaliase in phenylketonuria [43], allergy to elosulfase alfa in the Morquio syndorme [44] and allergy to idursulfase [45] and laronidase [9].

Importantly, in virtually all these cases, the time between each step was at least 15 minutes, as in our case. This interval time seems to be crucial in desensitization procedures, in order to avoid major adverse reactions. *In vitro* models of basophils desensitization demonstrated that human basophil from allergic patients, when repeatedly incubated with suboptimal doses of the allergen, reached a maximal unresponsiveness when the incubation time was between 15 and 30 min ~~[46,47]~~ [12,46].

In the ~~case~~ cases presented, the skin tests were positive, suggesting the allergic nature of the adverse reactions occurred.

Thus, rapid desensitization effectively ~~allowed~~ allows resumption of therapy, as in our three cases. But this procedure still has limits. Firstly, it requires a longer infusion time (up to 6 h), compared to the already long (up to 3 h) standard schedule, also considering that ERT should be continued indefinitely. Secondly, as in our cases, rapid desensitization to idursulfase and laronidase (both recombinant protein enzymes) were not exempt from occasional, often minor HSR, which, nevertheless, mandated enhanced premedication (including high-dose corticosteroids, not free from side effects) ~~[48]~~ [47].

With ~~the~~ relation to these limitations, we decided to manage our cases with a combination of rapid desensitization, resulting in resumption of ERT, and AIT-like desensitization, in order to induce a long-lasting immuno-tolerance ~~[49]~~ [48]. AIT has been known for many years to be effective in conferring tolerance for non-self protein enzymes, as in the case of Hymenoptera venom allergy ~~[50]~~ [49].

Thus, exploiting our previous experience with Hymenoptera venom AIT, we devised a compact AIT-like ~~desensitiation~~ desensitization protocol for idursulfase and laronidase, respectively. The two enzymes were administrated every second day subcutaneously, at increasing dosages, starting from approx. 8 μg (~~idurfulfase~~ idursulfase) and 50 μg (laronidase) at day-1, and reaching a dosage 500-fold higher, for idursulfase, and 222-fold higher, for laronidase, in about 24 days (11^th^ AIT session). This approach proved to be successful, allowing continuation of ERT in a standard infusion mode, with a single drug dilution (according to the drug’s leaflet). As in the case of Hymenoptera venom AIT, we performed monthly subcutaneous maintenance-phase injections, represented by the highest dosage reached in the induction course, at the 11^th^ session, *viz.* 4 mg for idursulfase and 2,000 U (11.6 mg) for laronidase.

Our approach of combined intravenous rapid desensitization and subcutaneous AIT-like desensitization is rather novel, to our knowledge, since only another case report could be found in the literature, regarding two breast cancer patients, desensitized for trastuzumab by a rapid ~~i.v.~~ intravenous protocol, with interspersed weekly subcutaneous administrations of the monoclonal antibody (20 mg), in order to control the still important hypersensitivity symptoms associated with the intravenous administration (2 mg/kg) in rapid desensitization mode [49].

In spite of the novelty, the combined approach appeared to be effective and safe, since it allowed restoration of ERT in a standard mode, minimizing the time required for the weekly infusion (down to approx. 3 h with a single dilution bag). This is not trivial in consideration of the fact that in patients with MPS who developed allergy to ERT, rapid desensitization in not free from HSR.

Therefore, we propose that this combined approach may be adopted in at least the more problematic cases of MPS with ERT allergy. By extension, this desensitization strategy could be used also in other conditions in which allergy to a protein drug has developed.

In contrast, as for hypersensitivity to platins and taxanes, which represent the major chemotherapeutic agents involved in the vast majority of allergy to cancer therapeutics, the AIT-like subcutaneous approach does not appear feasible, given the inherent cytotoxicity of these drugs and their different immunogenic (non-protein) profile.

**CONCLUSIONS**

To our knowledge, this is the first report showing the usefulness of rapid desensitization combined with an AIT-like desensitization for allergy to recombinant enzymes.

We propose to use this approach, which proved to be effective and safe, not only in patients with Hunter syndrome and Scheie syndrome, who develop HSR during ERT with idursulfase and laronidase, respectively, but also in ERT hypersensitivity in other types of MPS and in other rare diseases, being treated by recombinant ~~enzymes~~ enzyme medications. By extension, other forms of protein drug allergy (such as monoclonal antibody) might rely on this approach.

**LIST OF ABBREVIATIONS**

MPS: mucopolysaccharidosis

ERT: Enzyme replacement therapy

AIT-like: allergen immunotherapy-like

HSR: hypersensitivity reactions

**DECLARATIONS**

**Ethics approval and consent to participate**

Not applicable.

**Consent for publication**

Written informed consent was sought after and obtained from the ~~patient~~ patients, for publication. The work was carried out in compliance with the Declaration of Helsinki.

**Availability of data and material**

Data will be made available upon request.

**Competing interests**

The Authors declare that they have no conflicts of interest

**Funding**

The Authors declare that this study was carried out with institutional resources only.

**Authors' contributions**

F.S. and F.V. performed the allergic workup and carried out the desensitization procedure, with the help of D.G.M. and V.R.; ~~FS~~ F.S. wrote the manuscript; A.T. and P.S. carried out part of the clinical work and contributed with relevant ideas; M.P.R. and L.G. performed the laboratory assays; E.N., D.D.B., C.S., M.F.C. and Fr.Sa.~~,~~ provided ideas and critical reading of the manuscript; R.F. shared the responsibility of the work and revised the manuscript; L.M. conceived the desensitization approach, corrected the manuscript and secured the financial support. All authors have read and approved the manuscript.

**~~Aknowledgment~~ Acknowledgement**

Not applicable.

**Table 1**. Rapid desensitization protocol for idursulfase.

| **step** | **solution** | **minutes** | **rate (ml/h)** | **delivered ml** | **delivered dose (mg)** |
| --- | --- | --- | --- | --- | --- |
| **1** | 1/100 | 15 | 4 | 1 | 0.00048 |
| **2** | 1/100 | 15 | 10 | 2.5 | 0.0012 |
| **3** | 1/100 | 15 | 20 | 5 | 0.0024 |
| **4** | 1/100 | 15 | 40 | 10 | 0.0048 |
| **5** | 1/10 | 15 | 10 | 2.5 | 0.012 |
| **6** | 1/10 | 15 | 20 | 5 | 0.024 |
| **7** | 1/10 | 15 | 40 | 10 | 0.048 |
| **8** | 1/10 | 15 | 80 | 20 | 0.096 |
| **9** | 1/1 | 15 | 20 | 5 | 0.24 |
| **10** | 1/1 | 15 | 40 | 10 | 0.48 |
| **11** | 1/1 | 15 | 80 | 20 | 0.96 |
| **12** | 1/1 | 182 | 150 | 455 | 21.84 |
| **total** |  | 347 |  |  | 23.71 |

Target dose: ~~24mg~~ 24 mg. Dilution 1/100 (bag 1): 10 ml of dilution 1/10-bag 2 in 90 ml of saline (0.00048 mg/ml). Dilution 1/10 (bag 2): 10 ml of dilution 1/1-bag 3 in 90 ml of saline (0.0048 mg/ml). Dilution 1/1 (mother solution; bag 3): 24 mg of idursulfase in 500 ml of saline (0.048 mg/ml).

**Table 2.** Rapid desensitization protocol for laronidase.

| **step** | **solution** | **minutes** | **rate (ml/h)** | **delivered ml** | **delivered dose (mg)** |
| --- | --- | --- | --- | --- | --- |
| **1** | 1/100 | 15 | 4 | 1 | 0.00079 |
| **2** | 1/100 | 15 | 10 | 2.5 | 0.00197 |
| **3** | 1/100 | 15 | 20 | 5 | 0.00394 |
| **4** | 1/100 | 15 | 40 | 10 | 0.0079 |
| **5** | 1/10 | 15 | 10 | 2.5 | 0.0197 |
| **6** | 1/10 | 15 | 20 | 5 | 0.0394 |
| **7** | 1/10 | 15 | 40 | 10 | 0.079 |
| **8** | 1/10 | 15 | 80 | 20 | 0.158 |
| **9** | 1/1 | 15 | 20 | 5 | 0.394 |
| **10** | 1/1 | 15 | 40 | 10 | 0.79 |
| **11** | 1/1 | 15 | 80 | 20 | 1.58 |
| **12** | 1/1 | 182 | 150 | 455 | 35.89 |
| **total** |  | 347 |  |  | 38.96 |

Target dose: 39.44 mg (6,800 Units). Dilution 1/100 (bag 1): 10 ml of dilution 1/10-bag 2 in 90 ml of saline (0.00079 mg/ml). Dilution 1/10 (bag 2): 10 ml of dilution 1/1-bag 3 in 90 ml of saline (0.0079 mg/ml). Dilution 1/1 (mother solution; bag 3): 39.44 mg of laronidase in 500 ml of saline (0.079 mg/ml).

**Table 3**. AIT-like desensitization protocol for idursulfase.

| **step** | **inj # 1** | | | **inj # 2** | | | **inj # 3** | | | | **inj # 4** | | | ***total (mg)** |
| --- | --- | --- | --- | --- | --- | --- | --- | --- | --- | --- | --- | --- | --- | --- |
|  | **conc. (mg/ml)** | **vol.**  **(ml)** | **dose**  **(mg)** | **conc. (mg/ml)** | **vol.**  **(ml)** | **dose**  **(mg)** | **conc. (mg/ ml)** | **vol.**  **(ml)** | **dose**  **(mg)** | **conc.**  **(mg/ml)** | | **vol.**  **(ml)** | **dose**  **(mg)** |  |
| **1** | 0.02 | 0.1 | 0.002 | 0.02 | 0.1 | 0.002 | 0.02 | 0.2 | 0.004 |  | |  |  | 0.008 |
| **2** | 0.02 | 0.3 | 0.006 | 0.02 | 0.3 | 0.006 | 0.02 | 0.3 | 0.006 |  | |  |  | 0.018 |
| **3** | 0.2 | 0.15 | 0.03 | 0.2 | 0.15 | 0.03 | 0.2 | 0.3 | 0.06 |  | |  |  | 0.12 |
| **4** | 0.2 | 0.3 | 0.06 | 0.2 | 0.3 | 0.06 | 0.2 | 0.6 | 0.12 |  | |  |  | 0.24 |
| **5** | 2 | 0.15 | 0.3 | 2 | 0.15 | 0.3 | 2 | 0.15 | 0.3 | 2 | | 0.15 | 0.3 | 1.2 |
| **6** | 2 | 0.25 | 0.5 | 2 | 0.25 | 0.5 | 2 | 0.25 | 0.5 | 2 | | 0.25 | 0.5 | 2 |
| **7** | 2 | 0.5 | 1 | 2 | 0.5 | 1 | 2 | 0.5 | 1 | 2 | | 0.5 | 1 | 4 |
| **8** | 2 | 0.5 | 1 | 2 | 0.5 | 1 | 2 | 0.5 | 1 | 2 | | 0.5 | 1 | 4 |
| **9** | 2 | 0.5 | 1 | 2 | 0.5 | 1 | 2 | 0.5 | 1 | 2 | | 0.5 | 1 | 4 |
| **10** | 2 | 0.5 | 1 | 2 | 0.5 | 1 | 2 | 0.5 | 1 | 2 | | 0.5 | 1 | 4 |
| **11** | 2 | 0.5 | 1 | 2 | 0.5 | 1 | 2 | 0.5 | 1 | 2 | | 0.5 | 1 | 4 |

Total dose administered over ~~approx..~~ approx. 24 days: 23.59 mg. Inj #1 – Inj #4: at each step/session 3 or 4 subcutaneous injections were given, ~~20 ‘ apart~~ 20’apart from each other. *Total dose administered at each step (consisting of 3-4 subcutaneous injections).

**Table 4**. AIT-like desensitization protocol for laronidase.

| **step** | **inj # 1** | | | **inj # 2** | | | **inj # 3** | | | **inj # 4** | | | ***total**  **(U)** |
| --- | --- | --- | --- | --- | --- | --- | --- | --- | --- | --- | --- | --- | --- |
|  | **conc. (U/ml)** | **vol.**  **(ml)** | **U** | **conc. (U/ml)** | **vol.**  **(ml)** | **U** | **conc. (U/ml)** | **vol.**  **(ml)** | **U** | **conc.**  **(U/ml)** | **vol.**  **(ml)** | **U** |  |
| **1** | 10 | 0.01 | 1 | 10 | 0.02 | 2 | 10 | 0.03 | 3 | 10 | 0.03 | 3 | 9 |
| **2** | 10 | 0.03 | 3 | 10 | 0.06 | 6 | 10 | 0.06 | 6 | 10 | 0.06 | 6 | 21 |
| **3** | 100 | 0.1 | 10 | 100 | 0.1 | 10 | 100 | 0.15 | 15 | 100 | 0.15 | 15 | 50 |
| **4** | 100 | 0.15 | 15 | 100 | 0.15 | 15 | 100 | 0.3 | 30 | 100 | 0.6 | 60 | 120 |
| **5** | 100 | 1 | 100 | 100 | 1 | 100 | 100 | 1 | 100 | 100 | 1 | 100 | 400 |
| **6** | 100 | 1 | 100 | 100 | 1 | 100 | 500 | 0.4 | 200 | 500 | 0.4 | 200 | 600 |
| **7** | 500 | 0.4 | 200 | 500 | 0.4 | 200 | 500 | 0.6 | 300 | 500 | 1 | 500 | 1,200 |
| **8** | 500 | 1 | 500 | 500 | 1 | 500 | 500 | 1 | 500 | 500 | 1 | 500 | 2,000 |
| **9** | 500 | 1 | 500 | 500 | 1 | 500 | 500 | 1 | 500 | 500 | 1 | 500 | 2,000 |
| **10** | 500 | 1 | 500 | 500 | 1 | 500 | 500 | 1 | 500 | 500 | 1 | 500 | 2,000 |
| **11** | 500 | 1 | 500 | 500 | 1 | 500 | 500 | 1 | 500 | 500 | 1 | 500 | 2,000 |

Total dose administered over approx. 24 days: 10,400 U (60.32 mg). Inj #1 – Inj #4: at each step/session 3 or 4 subcutaneous injections were given, ~~20 ‘ apart~~ 20’apart

from each other. *Total dose administered at each step (consisting of 3-4 subcutaneous injections). At steps 1 and 2, laronidase was used diluted 10-fold in saline. From step 6 (injections #3 and #4, only) to step 11, laronidase was used concentrated 5-fold by ultrafiltration (see above).

**REFERENCES**

1. Brooks DA. Alpha-L-iduronidase and enzyme replacement therapy for mucopolysaccharidosis I. Expert Opin Biol Ther. 2002;2:967-76.

2. Hampe CS, Eisengart JB, Lund TC, Orchard PJ, Swietlicka M, Wesley J, McIvor RS. Mucopolysaccharidosis type I: a review of the natural history and molecular pathology. Cells. 2020;9:1838.

3. Clarke LA. Mucopolysaccharidosis type I. In: Adam MP, Ardinger HH, Pagon RA, et al., editors. GeneReviews® [Internet]. Seattle (WA): University of Washington; 2002. ~~P~~ p. 1993-2022.

4. D'Avanzo F, Rigon L, Zanetti A, Tomanin R. Mucopolysaccharidosis type II: one hundred years of research, diagnosis, and treatment. Int J Mol Sci. 2020;21:1258.

5. Wraith JE, Scarpa M, Beck M, Bodamer OA, De Meirleir L, Guffon N, Meldgaard Lund A, Malm G, Van der Ploeg AT, Zeman J. Mucopolysaccharidosis type II (Hunter syndrome): a clinical review and recommendations for treatment in the era of enzyme replacement therapy. Eur J Pediatr. 2008;167:267-77.

6. Muenzer J, Gucsavas-Calikoglu M, McCandless SE, Schuetz TJ, Kimura A. A phase I/II clinical trial of enzyme replacement therapy in mucopolysaccharidosis II (Hunter syndrome). Mol Genet Metab. 2007;90:329-37.

7. Dornelles AD, Artigalás O, da Silva AA, Ardila DLV, Alegra T, Pereira TV, Vairo FPE, Schwartz IVD. Efficacy and safety of intravenous laronidase for mucopolysaccharidosis type I: a systematic review and meta-analysis. PLoS One. 2017;12:e0184065.

8. Muenzer J, Beck M, Eng CM, Giugliani R, Harmatz P, Martin R, Ramaswami U, Vellodi A, Wraith JE, Cleary M, Gucsavas-Calikoglu M, Puga AC, Shinawi M, Ulbrich B, Vijayaraghavan S, Wendt S, Conway AM, Rossi A, Whiteman DA, Kimura A. Long-term, open-labeled extension study of idursulfase in the treatment of Hunter syndrome. Genet Med. 2011;13:95-101.

9. Ensina LF, Aranda CS, de Lacerda AE, Camelo-Nunes I, Sole D, Martins AM, Castells M. Laronidase hypersensitivity and desensitization in type I mucopolysaccharidosis: a case report. Pediatr Allergy Immunol. 2014;25:498-9.

10. Bustamante LL, Garavaglia L, Garramone EI, Amartino H, Parisi CA. Desensibilización con idursulfase en un niño con síndrome de Hunter (mucopolisacaridosis II) [Idursulfase desensitization in a child with Hunter syndrome (mucopolysaccharidosis II)]. Arch Argent Pediatr. 2021;119:e41-e44. Spanish.

11. Karimian Z,Whitley CB, Rudser KD, Jarnes-Utz JR. Delayed infusion reactions to enzyme replacement therapies. JIMD Rep. 2017;34:63-70.

12. Cernadas JR, Brockow K, Romano A, Aberer W, Torres MJ, Bircher A, Campi P, Sanz ML, Castells M, Demoly P, Pichler WJ; European Network of Drug Allergy and the EAACI interest group on drug hypersensitivity. General considerations on rapid desensitization for drug hypersensitivity - a consensus statement. Allergy. 2010;65:1357-66.

13. Castells MC, Tennant NM, Sloane DE, Hsu FI, Barrett NA, Hong DI, Laidlaw TM, Legere HJ, Nallamshetty SN, Palis RI, Rao JJ, Berlin ST, Campos SM, Matulonis UA. Hypersensitivity reactions to chemotherapy: outcomes and safety of rapid desensitization in 413 cases. J Allergy Clin Immunol. 2008;122:574-80.

14. Passalacqua G, Canonica GW. Allergen ~~Immunotherapy~~ immunotherapy: history and future developments. Immunol Allergy Clin North Am. 2016;36:1-12.

15. Wahn U, Bachert C, Heinrich J, Richter H, Zielen S. Real-world benefits of allergen immunotherapy for birch pollen-associated allergic rhinitis and asthma. Allergy. 2019;74:594-604.

16. Kim JY, Jang MJ, Kim DY, Park SW, Han DH. Efficacy of subcutaneous and sublingual immunotherapy for house dust mite allergy: ~~A~~ a network meta-analysis-based comparison. J Allergy Clin Immunol Pract. 2021;9:4450-8.

17. Bilò MB, Pravettoni V, Bignardi D, Bonadonna P, Mauro M, Novembre E, Quercia O, Cilia M, Cortellini G, Costantino MT, Cremonte L, Lodi Rizzini F, Macchia L, Marengo F, Murzilli F, Patella V, Reccardini F, Ricciardi L, Ridolo E, Romano A, Savi E, Schiavino D, Severino M, Pastorello EA. Hymenoptera venom allergy: management of children and adults in clinical practice. J Investig Allergol Clin Immunol. 2019;29:180-205.

18. Kołaczek A, Skorupa D, Antczak-Marczak M, Kuna P, Kupczyk M. Safety and efficacy of venom immunotherapy: a real life study. Postepy Dermatol Alergol. 2017;34:159-67.

19. Albanesi M, Nico A, Sinisi A, Giliberti L, Rossi MP, Rossini M, Kourtis G, Rucco AS, Loconte F, Muolo L, Zurlo M, Di Bona D, Caiaffa MF, Macchia L. A 13-year real-life study on efficacy, safety and biological effects of *Vespula* venom immunotherapy. Clin Mol Allergy. 2018;16:2.

20. Leader BA, Rotella M, Stillman L, DelGaudio JM, Patel ZM, Wise SK. Immunotherapy compliance: comparison of subcutaneous versus sublingual immunotherapy. Int Forum Allergy Rhinol. 2016;6:460-4.

21. Chaoul N, Albanesi M, Giliberti L, Rossi MP, Nettis E, Di Bona D, Caiaffa MF, Macchia L. Maintenance-phase subcutaneous immunotherapy with house dust mites induces cyclic immunologic effects. Int Arch Allergy Immunol. 2019;179:37-42.

22. Bilò BM, Bonifazi F. Hymenoptera venom immunotherapy. Immunotherapy. 2011;3:229-46.

23.<https://www.ema.europa.eu/en/documents/product-information/elaprase-epar-product-information_en.pdf>. Accessed August 1, 2022.

24. <https://www.ema.europa.eu/en/documents/product-information/aldurazyme-epar-product-information_en.pdf>. Accessed August 1, 2022.

25. Corallino M, Nico A, Kourtis G, Caiaffa MF, Macchia L. Skin testing technique and precision in stinging insect allergy. J Clin Nurs. 2007;16:1256-64.

26. ~~Elena Minenna, Nada Chaoul, Maria Pia Rossi, Lucia Giliberti, Marcello Albanesi, Eustachio Nettis, Maria Pia Foschino Barbaro, Danilo Di Bona, Maria Filomena Caiaffa, Luigi Macchia~~ Minenna E, Chaoul N, Rossi MP, Giliberti L, Albanesi M, Nettis E, Foschino Barbaro MP, Di Bona D, Caiaffa MF, Macchia L. Sustained drug-related reaction with eosinophilia and systemic symptoms (DRESS) triggered by low molecular weight heparins in COVID-19: management and precision diagnosis. Adv Dermatol Allergol DOI: https://doi.org/10.5114/ada.2021.109586.

27. Xie H, Chung JK, Mascelli MA, McCauley TG. Pharmacokinetics and bioavailability of a therapeutic enzyme (idursulfase) in cynomolgus monkeys after intrathecal and intravenous administration. PLoS One. 2015;10:e0122453.

28. Heil J, Reifferscheid G. Detection of mammalian carcinogens with an immunological DNA synthesis-inhibition test. Carcinogenesis. 1992;13:2389-94.

29. Porebski G, Gschwend-Zawodniak A, Pichler WJ. In vitro diagnosis of T cell-mediated drug allergy. Clin Exp Allergy. 2011;41:461-70.

30. Albanesi M, Di Girolamo A, Aresta V, Rossi MP, Giliberti L, Perrone T, Di Bona D, Caiaffa MF, Specchia G, Macchia L. Delayed allergy to acyclovir revealed by lymphocyte proliferation test. Postepy Dermatol Alergol. 2018;35:527-9.

31. Burton BK, Whiteman DA; HOS Investigators. Incidence and timing of infusion-related reactions in patients with mucopolysaccharidosis type II (Hunter syndrome) on idursulfase therapy in the real-world setting: a perspective from the Hunter Outcome Survey (HOS). Mol Genet Metab. 2011;103:113-20.

32. Clarke LA, Wraith JE, Beck M, Kolodny EH, Pastores GM, Muenzer J, Rapoport DM, Berger KI, Sidman M, Kakkis ED, Cox GF. Long-term efficacy and safety of laronidase in the treatment of mucopolysaccharidosis I. Pediatrics. 2009;123:229-40.

33. Fica A, Muñoz D, Rojas T, Sanzana C, Muñoz C. Penicillin desensitization in allergic pregnant women with syphilis. Report of two cases. Rev Med Chil. 2020;148:344-8.

34. Garcia JFB, Aun MV, Motta AA, Castells M, Kalil J, Giavina-Bianchi P. Algorithm to guide re-exposure to penicillin in allergic pregnant women with syphilis: Efficacy and safety. World Allergy Organ J. 2021;14:100549.

35. Coop CCA, Berg MJR. Rapid ~~Desensitization~~ desensitization after a type I hypersensitivity reaction to ceftazidime/ avibactam. Fed Pract. 2022;39:94-6.

36. Staso P, Leonov A. Drug desensitization in 17-year-old male with ~~Mast Cell Activation~~ ~~Syndrome~~ mast cell activation syndrome, pneumonia, and antibiotic hypersensitivities. AME Case Rep. 2017;1:7.

37. Makowska J, Lewandowska-Polak A. Desensitization to biological agents used in rheumatology. Reumatologia. 2020;58:25-33.

38. Cansever M, Şahin N, Dursun I, Geyik C, Düşünsel R, Bektaş Kut F, Tahan F. Successful slow desensitization to tocilizumab in a 15-year-old patient. J Investig Allergol Clin Immunol. 2018;28:436-8.

39. Behera SK, Selvarajan S, Mathews J, Shajil C, Das S, Munisamy M, Xavier AS. A ~~Novel~~ novel desensitization protocol for infliximab hypersensitivity in a 13-year old patient with pustular psoriasis. Curr Drug Saf. 2019;14:158-62.

40. de Lira-Quezada CE, Villarreal-González RV, González-Díaz SN, Acuña-Ortega N. Protocol for desensitization to trastuzumab in a patient with anaphylaxis and stage IV breast cancer: a case report. J Investig Allergol Clin Immunol. 2020;30:376-7.

41. Di Girolamo A, Albanesi M, Loconte F, Di Bona D, Caiaffa MF, Macchia L. Desensitization in iron product allergy. Acta Haematol. 2020;143:496-9.

42. Rodríguez-Jiménez B, Domínguez-Ortega J, Nuñez-Acevedo B, Cava-Sumner B, Kindelan-Recarte C, Montojo-Guillén C. Rapid iron desensitization after generalized urticaria and facial angioedema. J Investig Allergol Clin Immunol. 2014;24:69-71.

43. Patrawala M, Kuruvilla M, Li H. Successful desensitization of Pegvaliase (Palynziq®) in a patient with phenylketonuria. Mol Genet Metab Rep. 2020;23:100575.

44. Guvenir H, Dibek Misirlioglu E, Capanoglu M, Buyuktiryaki B, Unal O, Toyran M, Kocabas CN. Successful desensitization of elosulfase alfa-induced anaphylaxis in a pediatric patient with Morquio syndrome. J Allergy Clin Immunol Pract. 2017;5:1156-7.

45. Serrano CD, Gomez JF. Successful desensitization to idursulfase in a patient with type II mucopolysaccharidosis (Hunter syndrome). J Investig Allergol Clin Immunol. 2011;21:571-2.

46. Pienkowski MM, Kazmier WJ, Adkinson NF Jr. Basophil histamine release remains unaffected by clinical desensitization to penicillin. J Allergy Clin Immunol. 1988;82:171-8.

~~47. Cernadas JR, Brockow K, Romano A, Aberer W, Torres MJ, Bircher A, Campi P, Sanz ML, Castells M, Demoly P, Pichler WJ; European Network of Drug Allergy and the EAACI interest group on drug hypersensitivity. General considerations on rapid desensitization for drug hypersensitivity - a consensus statement. Allergy. 2010;65:1357-66.~~

~~48. Rosenberg J, Jhaveri P, Kelbel T. Laronidase desensitization during stem cell transplant in a child with Hurler syndrome. Ann Allergy Asthma Immunol. 2016;116:377-8.~~

47. Rosenberg J, Jhaveri P, Kelbel T. Laronidase desensitization during stem cell transplant in a child with Hurler syndrome. Ann Allergy Asthma Immunol. 2016;116:377-8

~~49. Melamed J, Stahlman JE. Rapid desensitization and rush immunotherapy to trastuzumab (Herceptin). J Allergy Clin Immunol. 2002;110:813-4.~~

48. Melamed J, Stahlman JE. Rapid desensitization and rush immunotherapy to trastuzumab (Herceptin). J Allergy Clin Immunol. 2002;110:813-4.

~~50. Sturm GJ, Varga EM, Roberts G, Mosbech H, Bilò MB, Akdis CA, Antolín-Amérigo D, Cichocka-Jarosz E, Gawlik R, Jakob T, Kosnik M, Lange J, Mingomataj E, Mitsias DI, Ollert M, Oude Elberink JNG, Pfaar O, Pitsios C, Pravettoni V, Ruëff F, Sin BA, Agache I, Angier E, Arasi S, Calderón MA, Fernandez-Rivas M, Halken S, Jutel M, Lau S, Pajno GB, van Ree R, Ryan D, Spranger O, van Wijk RG, Dhami S, Zaman H, Sheikh A, Muraro A. EAACI guidelines on allergen immunotherapy: Hymenoptera venom allergy. Allergy. 2018;73:744-764.~~

49. Sturm GJ, Varga EM, Roberts G, Mosbech H, Bilò MB, Akdis CA, Antolín-Amérigo D, Cichocka-Jarosz E, Gawlik R, Jakob T, Kosnik M, Lange J, Mingomataj E, Mitsias DI, Ollert M, Oude Elberink JNG, Pfaar O, Pitsios C, Pravettoni V, Ruëff F, Sin BA, Agache I, Angier E, Arasi S, Calderón MA, Fernandez-Rivas M, Halken S, Jutel M, Lau S, Pajno GB, van Ree R, Ryan D, Spranger O, van Wijk RG, Dhami S, Zaman H, Sheikh A, Muraro A. EAACI guidelines on allergen immunotherapy: Hymenoptera venom allergy. Allergy. 2018;73:744-764.
